# Supplementary material for: TGFβ1+CCR5+ neutrophil subset increases in bone marrow and causes age-related osteoporosis in male mice
Source: Nat Commun. 2023 Jan 11;14:159. doi: 10.1038/s41467-023-35801-z (PMC9834218; doi:10.1038/s41467-023-35801-z)
Supplement: Supplementary file 1 — Supplementary Information [file 41467_2023_35801_MOESM1_ESM.pdf]

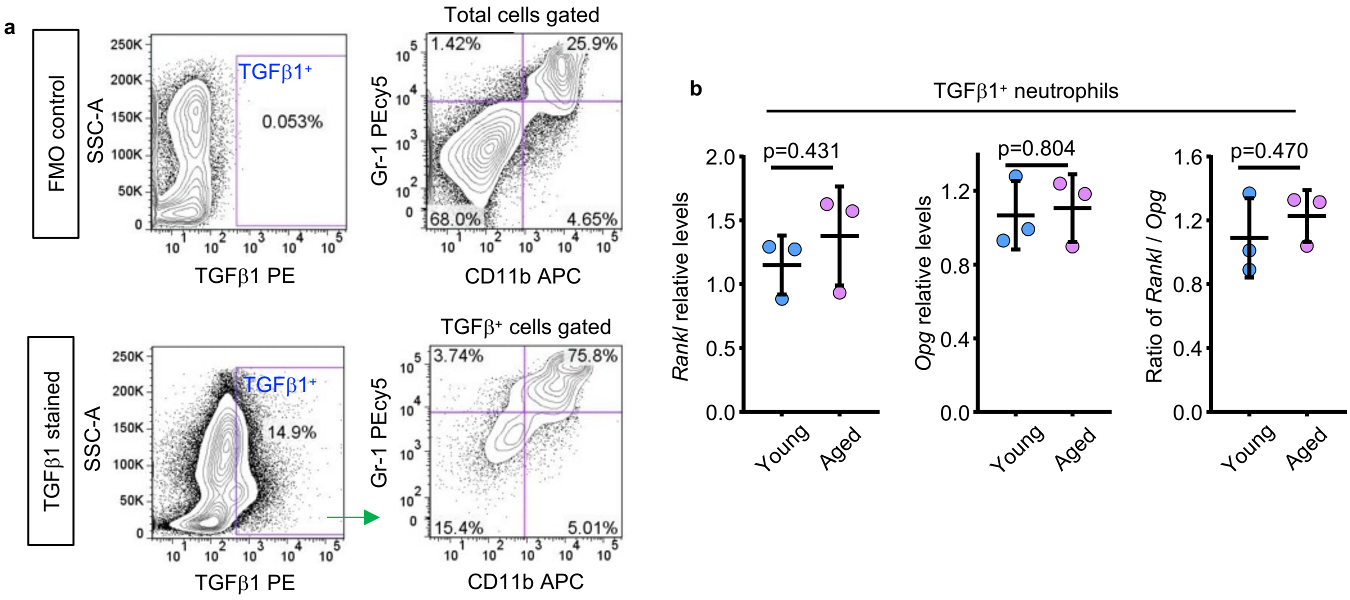

Supplementary Figure 1. Characterization of TGFβ1^{+} bone marrow cells. (**a**) Full minus one (FMO) control (Upper panel) and stained samples (lower panel) were gated for TGFβ1^{+}, CD11b^{+} and Gr1^{+} (Ly6C/6G) cell populations in BM cells using FACS. (**b**) Gene expression levels of *Rankl* and *Opg* were tested in TGFβ1^{+} neutrophils (CD11b^{+}TGFβ1^{+}Ly6C^{-}6G^{+}) cells sorted by FACS from 3-mon-old (Young) and 20-mon-old (Aged) C57 male mice. Mean±SD (n=3 biologically independent samples). Analyses in (**b**) done using Student's two-sided unpaired *t* test.

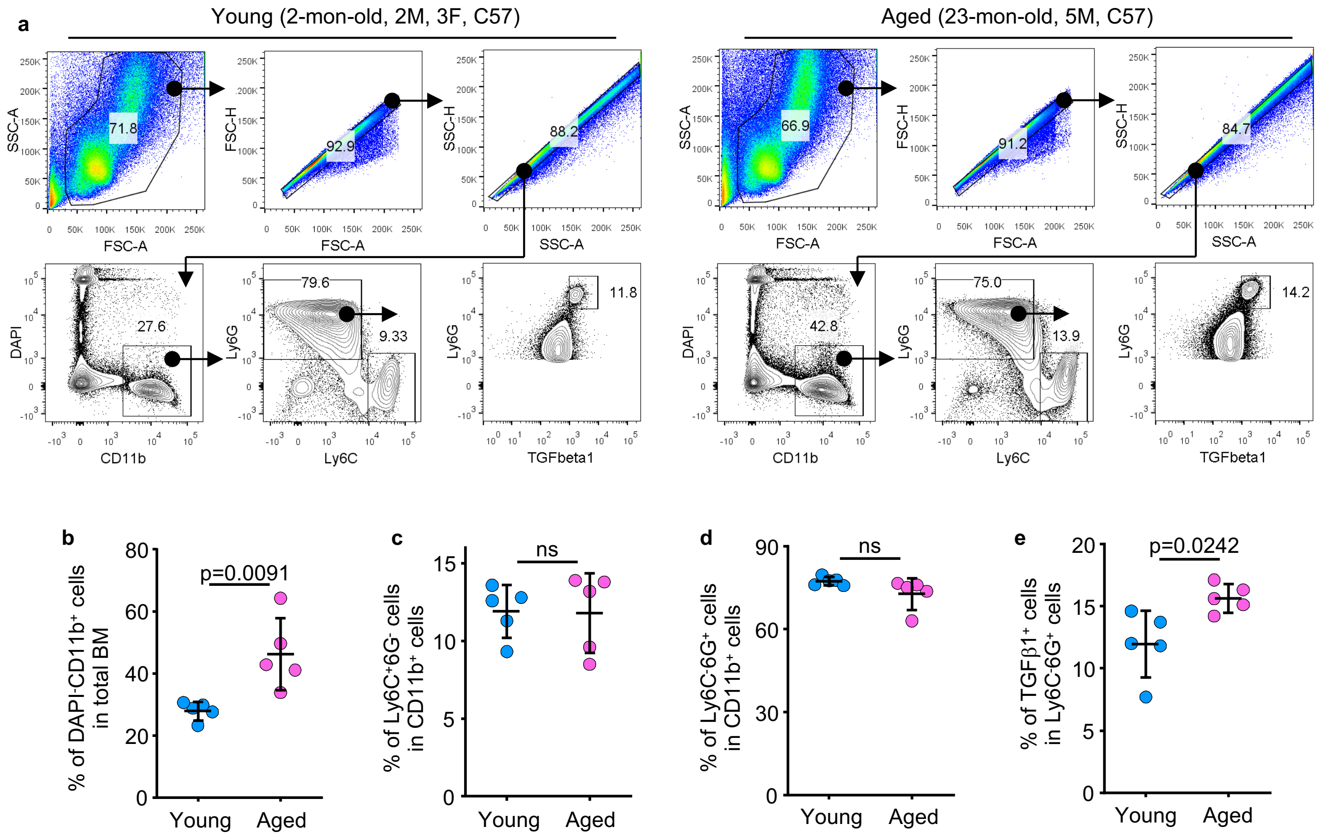

Supplementary Fig. 2. TGFβ1 expression by neutrophils. (a) Representative images showing flow gates for TGFβ1 expression in neutrophil population in BM cells from young and aged mice. (b) Frequencies of live leukocytes (DAPI-CD11b+) in total BM cells. Mean±SD (n=5 biologically independent male mice). (c-d) Frequencies of monocytic cells ($Ly6C^{+}6G^{-}$) (c) and neutrophils ($Ly6C^{-}6G^{+}$) (d) in leukocytes. Mean±SD (n=5 biologically independent male mice). (e) Frequencies of $TGF\beta1^{+}$ cells in neutrophils ($Ly6C^{-}6G^{+}$) in BM cells from young and aged mice. Mean±SD (n=5 biologically independent male mice). Analyses in (**b-e**) done using Student's two-sided unpaired *t* test.

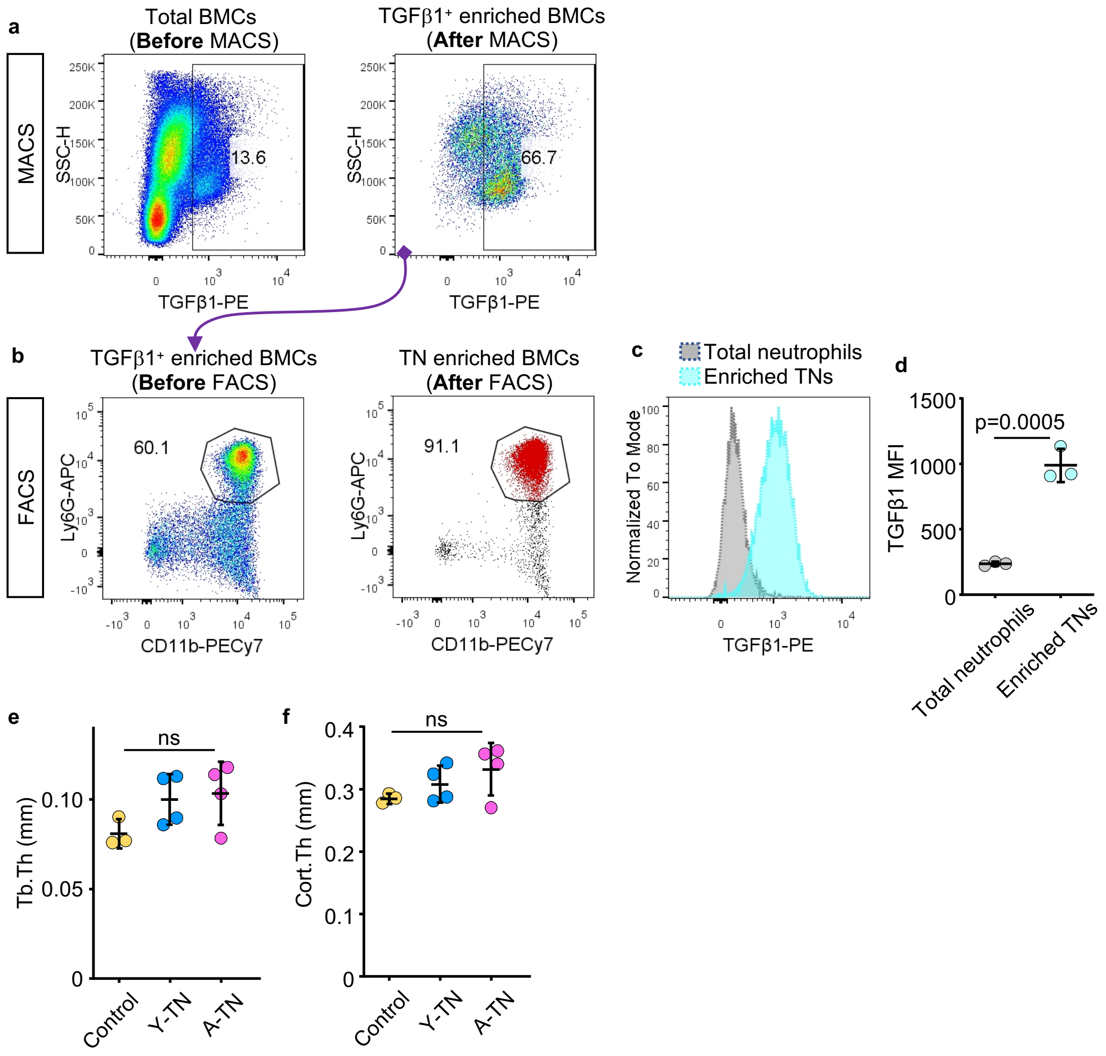

Supplementary Figure 3. TGFβ-expressing neutrophil isolation and transplantation. (**a**) TGFβ1 expression in total BM from 20-22-mon-old C57 male mice before magnetic-activated cell sorting (MACS) and TGFβ1$^+$ enriched BMCs after MACS. (**b**) TGFβ1$^+$-enriched BMCs after MACS further processed to sort CD11b$^+$Ly6G$^+$ neutrophils via fluorescence-activated cell sorting (FACS). The percentages of CD11b$^+$Ly6G$^+$ BMCS were analyzed in the samples before and after FACS. (**c-d**) Relative levels of TGFβ1 expression by total Ly6G$^+$ neutrophils (non-enriched) and sorted TGFβ1-expressing Ly6G$^+$ cells (TNs; enriched), (**c**) and mean of fluorescence intensity (MFI) (**d**). Mean±SD (n=3 biologically independent samples). **(e-f)** Trabecular (a) and cortical (b) bone thickness of NSG mice transplanted with TNs isolated from 3- and 20-22-mon-old C57 male mice. Mean±SD (n=3, 4 and 4 biologically independent male mice, respectively). Analyses in (**d**) done using unpaired Student's two-sided unpaired *t* test and in (**e** and **f**) using one-way ANOVA with Tukey's post-hoc test.

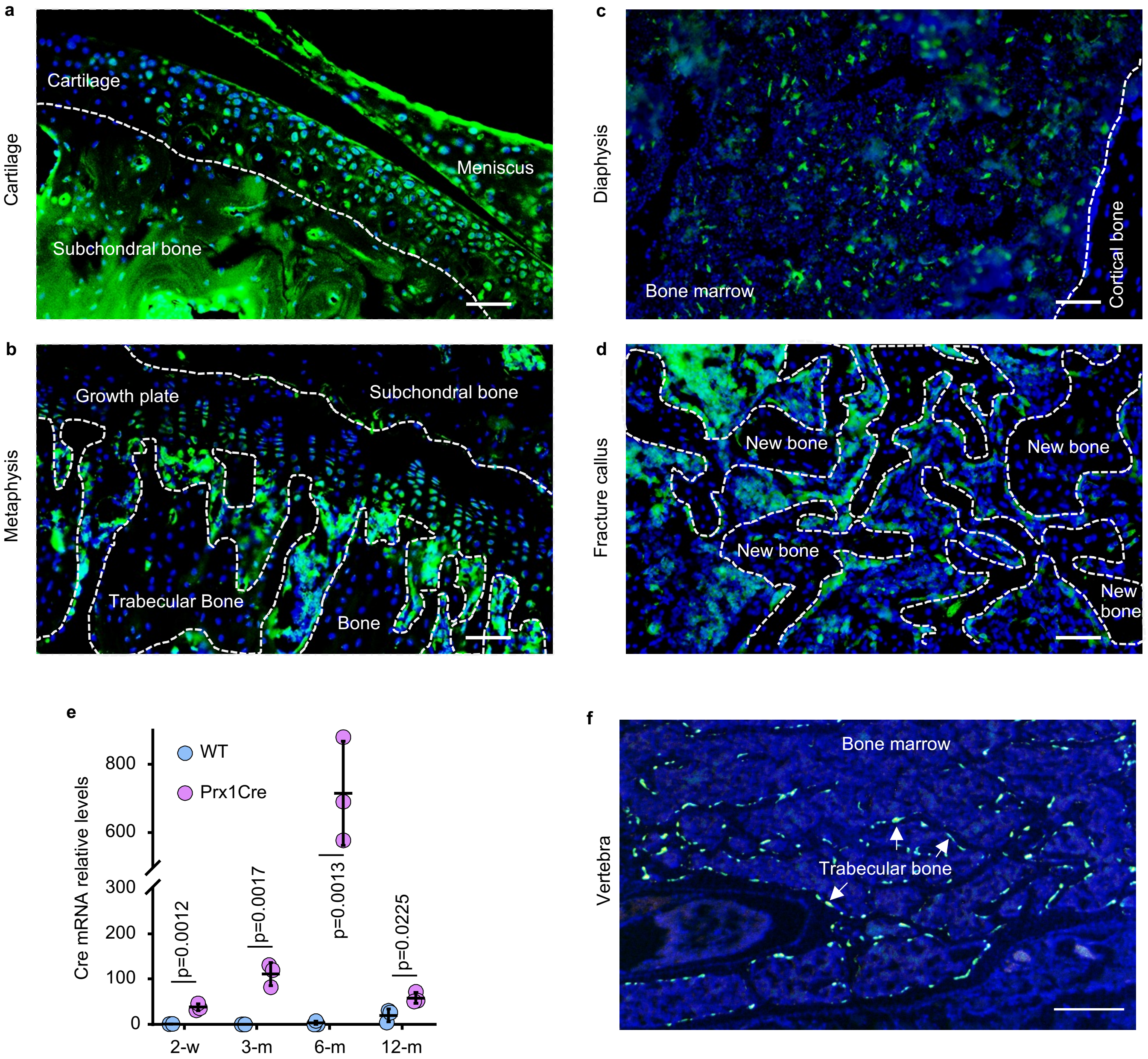

Supplementary Fig. 4. Verification of Prx1-driven Cre expression in bone cells via lineage tracing. (a-c) Cryosections of tibiae from 3-mon-old $Prx1^{Cre}$;$ROSA^{mTmG}$ mice co-stained with DAPI (blue) showing cartilage (a), metaphyseal (b), and diaphyseal (c) regions. Cells (green) with Cre expression driven by Prx1 promotor detected by GFP fluorescence. Bar=50μm. (d)) Cryosections of tibial fracture callus harvested from 3-mon-old $Prx1^{Cre}$;$ROSA^{mTmG}$ mice at day 14 post fracture. Osteoblastic cells (green) coating surfaces of newly generated bone detected by GFP fluorescence. Bar=50μm. (e) Cre mRNA relative expression levels of vertebral bone tissue from WT and $Prx1^{Cre}$ male mice aged 2 wk, 3, 6 and 12 mon. Mean±SD (n=3 biologically independent male mice). (f) Vertebral cryosection from 3-mon-old $Prx1^{Cre}$;$ROSA^{mTmG}$ mice co-stained with DAPI showing Cre-expressing cells (green) coating surfaces of trabecular bone. Bar=400μm. Analyses in (**e**) done using Student's two-sided unpaired *t* test.

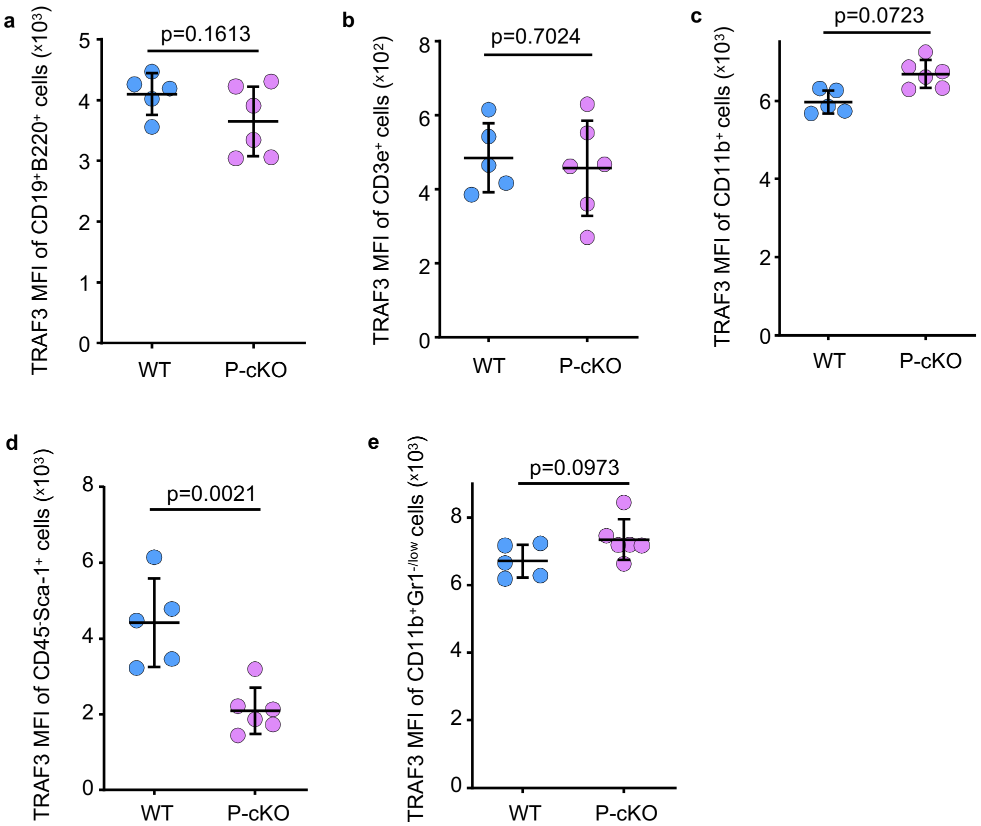

Supplementary Fig. 5. TRAF3 expression by various cell types in bone marrow of 15-mon-old TRAF3$^{f/f}$ and Prx1^{Cre};TRAF3$^{f/f}$ mice. (**a**-**e**) BM cells flushed from femora of 15-mon-old TRAF3$^{f/f}$ (WT) and Prx1^{Cre};TRAF3$^{f/f}$ (P-cKO) male mice and stained with Abs to various surface antigens, and intracellular TRAF3 protein. Mean fluorescence intensity (MFI) of TRAF3 expression (**a**) CD19^{+}B220^{+} B cells, (**b**) CD3e^{+} T cells, (**c**) CD11b^{+} leukocytes, (**d**) CD45^{-}Sca1^{+} mesenchymal progenitor cells and (**e**) CD11b^{+}Gr1$^{-/low}$ osteoclast precursor cells in BM. Mean±SD (n=5 and 6 biologically independent male mice for WT and P-cKO groups, respectively). Analyses: Student's two-sided unpaired *t* test.

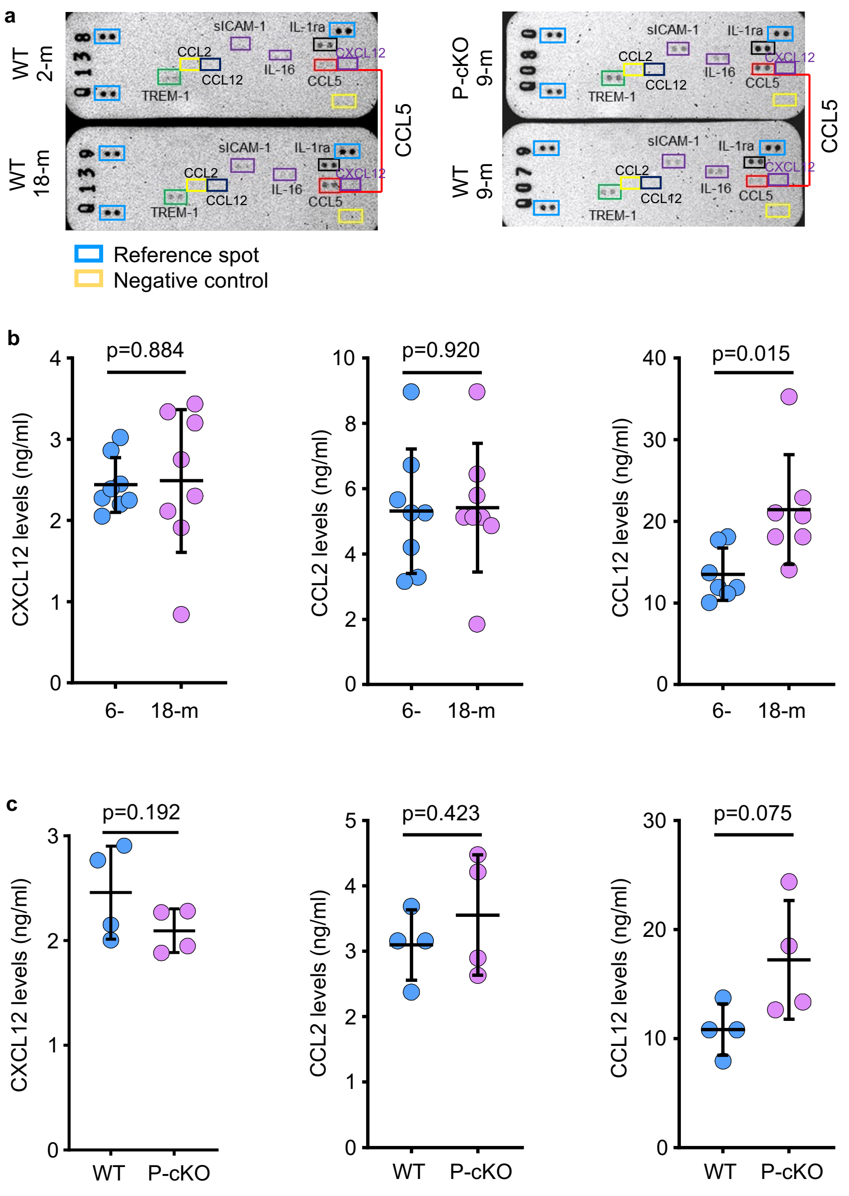

Supplementary Figure 6. Chemokine expression levels in bone marrow lysates. (a) Cytokine array of BM protein lysate from 2- and 18-mon-old male C57 mice and from 9-mon-old WT and P-cKO male mice. **(b)** Protein levels of CXCL12, CCL2 and CCL12 in BM protein lysate from 6- and 18-mon-old C57 mice measured using ELISA. Mean±SD (n=8, 8 and 7 biologically independent male mice per group in CXCL12, CCL2 and CCL12 assays, respectively). **(c)** Protein levels of CXCL12, CCL2 and CCL12 in BM lysates from 8-mon-old TRAF3$^{f/f}$ (WT) and Prx1^{Cre};TRAF3$^{f/f}$ (P-cKO) male mice measured using ELISA. Mean±SD (n=4 biologically independent male mice per group in all assays). Analyses in (**b** and **c**) done using Student's two-sided unpaired *t* test.

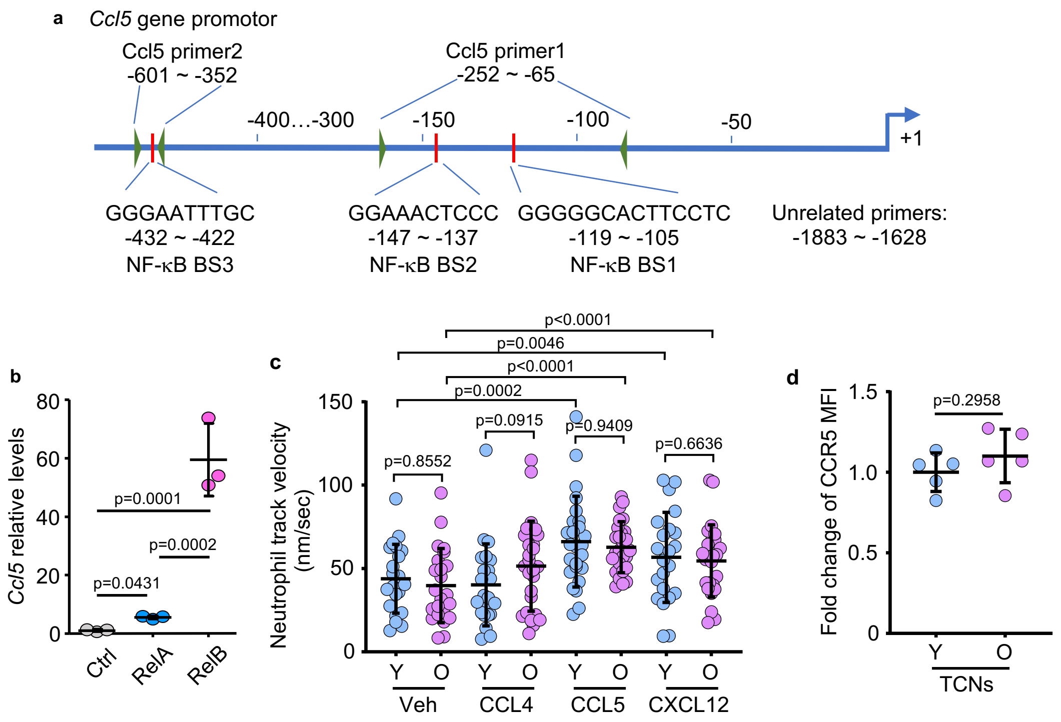

Supplementary Figure 7. NF-κB increases CCL5 expression by mesenchymal stromal cells and facilitates TCN motility. (**a**) Scheme for mouse Ccl5 promoter analysis showing putative κB binding sites and primer design to test binding sites (BS). (**b**) Calvarial pre-OBs from 7-d-old C57 mice infected with GFP control, RelA, or RelB retroviruses for 48 h. mRNA expression of *Ccl5* using real-time qPCR. Mean±SD (n=3 biologically independent samples/group). (**c**) Track velocity of neutrophils isolated from young and old C57 mice and in response to vehicle, CCL4, CCL5 or CXCL12. Mean±SD (n=22 biologically independent cells/group). (**d**) Fold change of CCR5 expression levels by TCNs from young and old C57 mice. Mean±SD (n=5 biologically independent male mice/group). Analyses in (**b** and **c**) done using One-way ANOVA with Tukey's post-hoc test and in (**d**) using Student's two-sided unpaired *t* test.

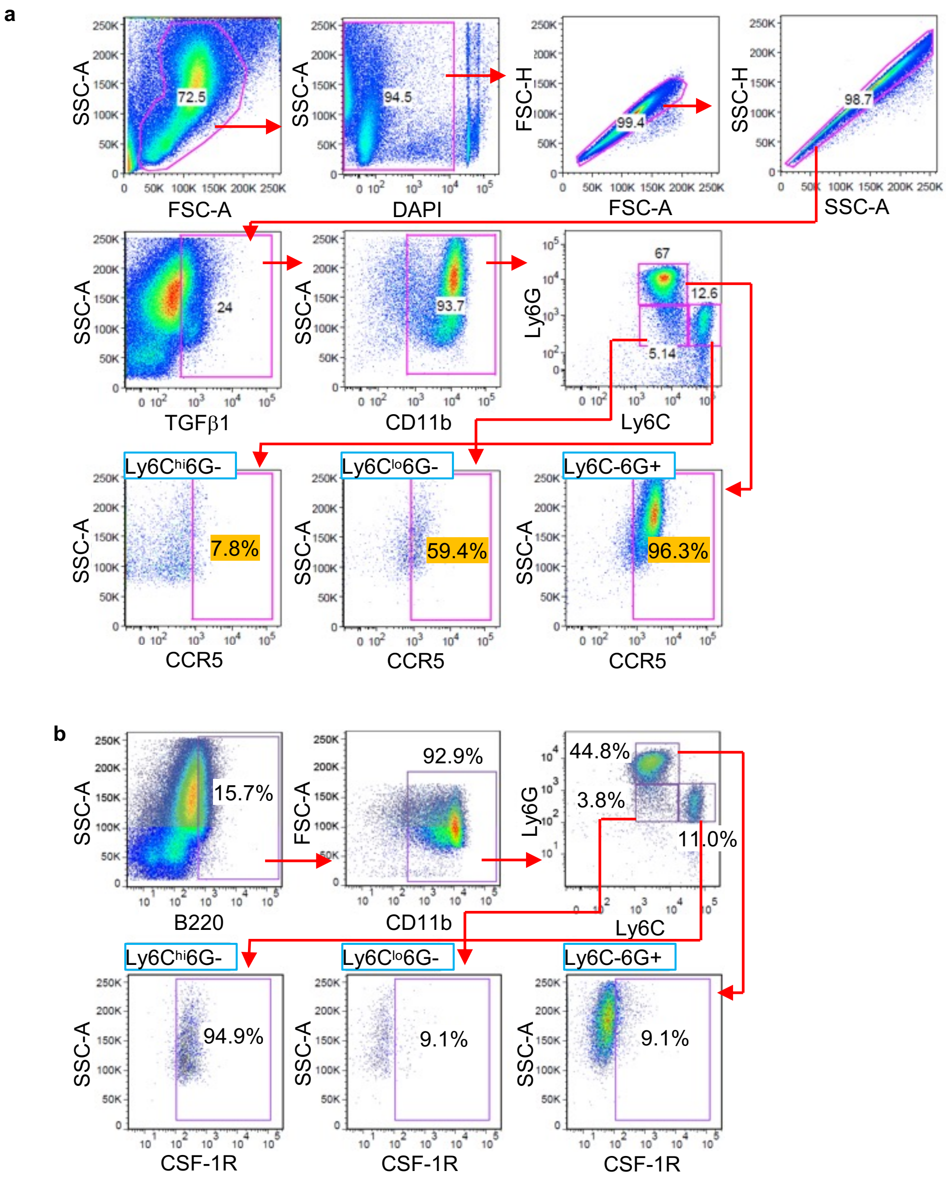

Supplementary Figure 8. CSF-1R and CCR5 expression by subpopulations of TGFβ1^{+}CD11b^{+} cells. (**a**) FACS gates of monocytic (Ly6C^{hi}6G^{-}), granulocytic (Ly6C^{-}6G^{+}) and intermediate-stage (Ly6C^{lo}6G^{-}) BM cells expressing intracellular TGFβ1 in BM of 22-mon-old C57 male mice and percentages of these cells expressing CCR5 (n=5 biologically independent male mice). (**b**) FACS gates of the subpopulations of TGFβ1^{+}CD11b^{+} cells in BM of 22-mon-old C57 mice, as in (a), and percentage of these cells expressing CSF-1R (M-CSF receptor) (n=8 biologically independent male mice).

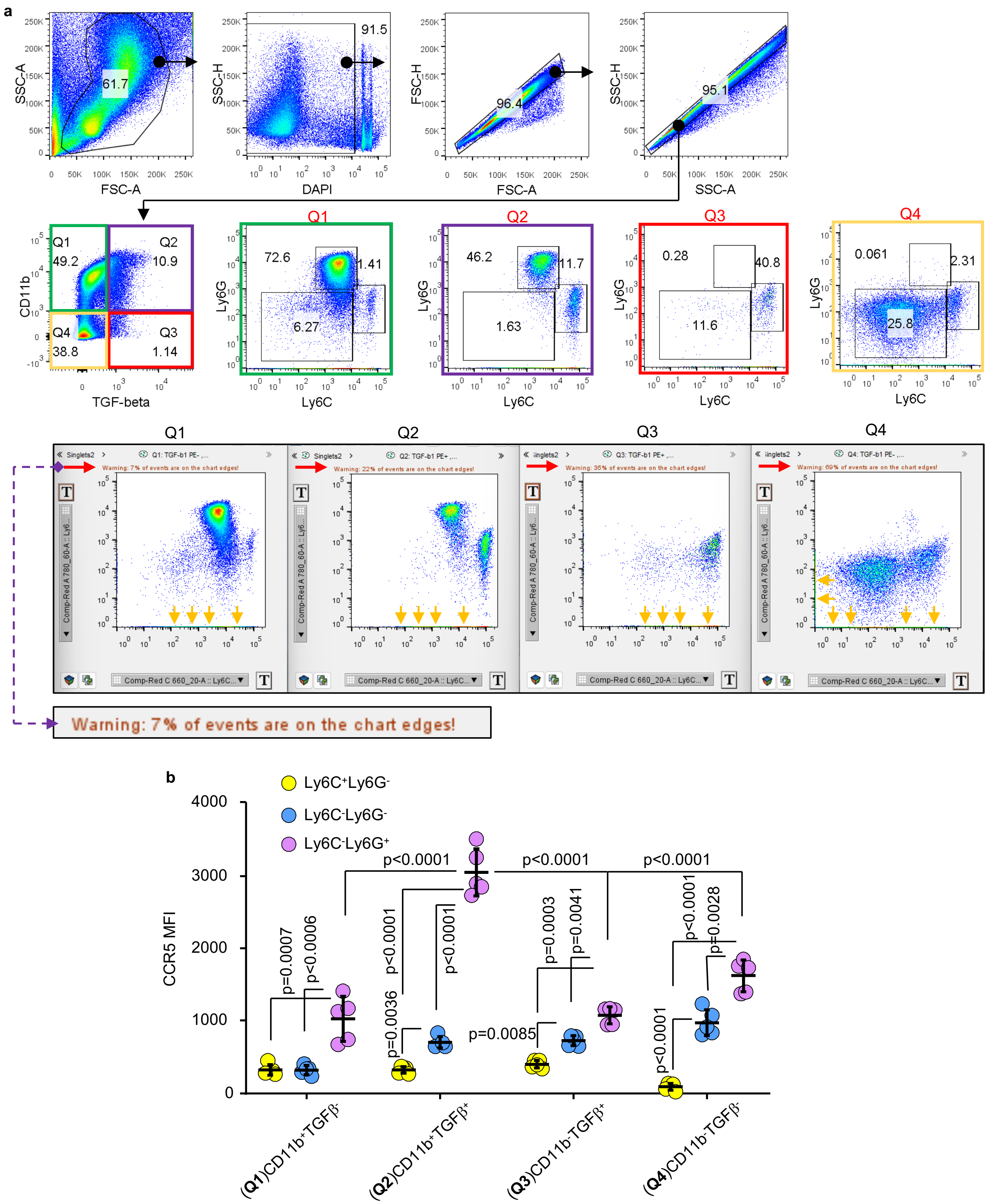

Supplementary Fig. 9. CCR5 expression by various $TGF\beta 1^-$ and $TGF\beta 1^+$ immune cell populations. (a) Representative images showing flow gates for leukocytes ($CD11b^+$) and non-leukocytes ($CD11b^-$) with or without TGFβ1 expression in BM from 23-mon-old C57 male mice. The numbers represent the percentages of the gated populations in all cells of each panel (the middle row). In Q1-4 panels, "log" axis was applied to show the clearly separated Ly6G and Ly6C subpopulations, and consequently various types of cells were hidden beyond the chart edges (indicated by the yellow arrows). (b) Mean fluorescence intensity of CCR5 expression by $Ly6C^+6G^-$, $Ly6C^-6G^-$ and $Ly6C^-6G^+$ subpopulation in leukocytes ($CD11b^+$) and non-leukocytes ($CD11b^-$) with or without TGFβ1 expression. Mean±SD (n=5 biologically independent male mice/group). Analyses in (**b**) done using Two-way ANOVA with multiple comparisons.

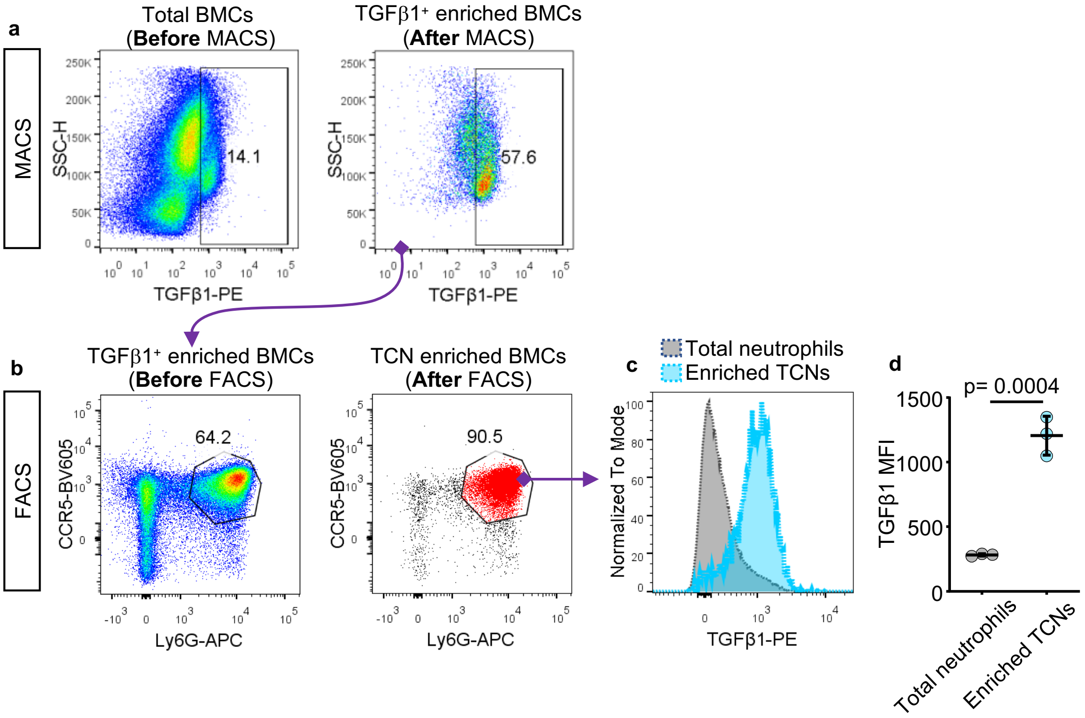

Supplementary Figure 10. Confirmation of TGFβ1 expression by sorted TCNs. (**a**) TGFβ1 expression by total BM from 20-22-mon-old ROSA^{mTmG} male mice before magnetic-activated cell sorting (MACS) and TGFβ1^{+}-enriched BMCs after MACS. (**b**) TGFβ1^{+}-enriched BMCs after MACS further processed to sort Ly6G and CD195 (CCR5) double-positive cells via fluorescence-activated cell sorting (FACS). Percentages of Ly6G^{+}CCR5^{+} BMCS analyzed in the samples before and after FACS. (**c-d**) Relative levels of TGFβ1 expression by total Ly6G^{+} neutrophils (non-enriched) and sorted TCNs (enriched), (**c**) and mean fluorescence intensity (MFI) (**d**). Mean±SD (n=3 biologically independent samples). Analyses in (**d**) done using Student's two-sided unpaired *t* test.

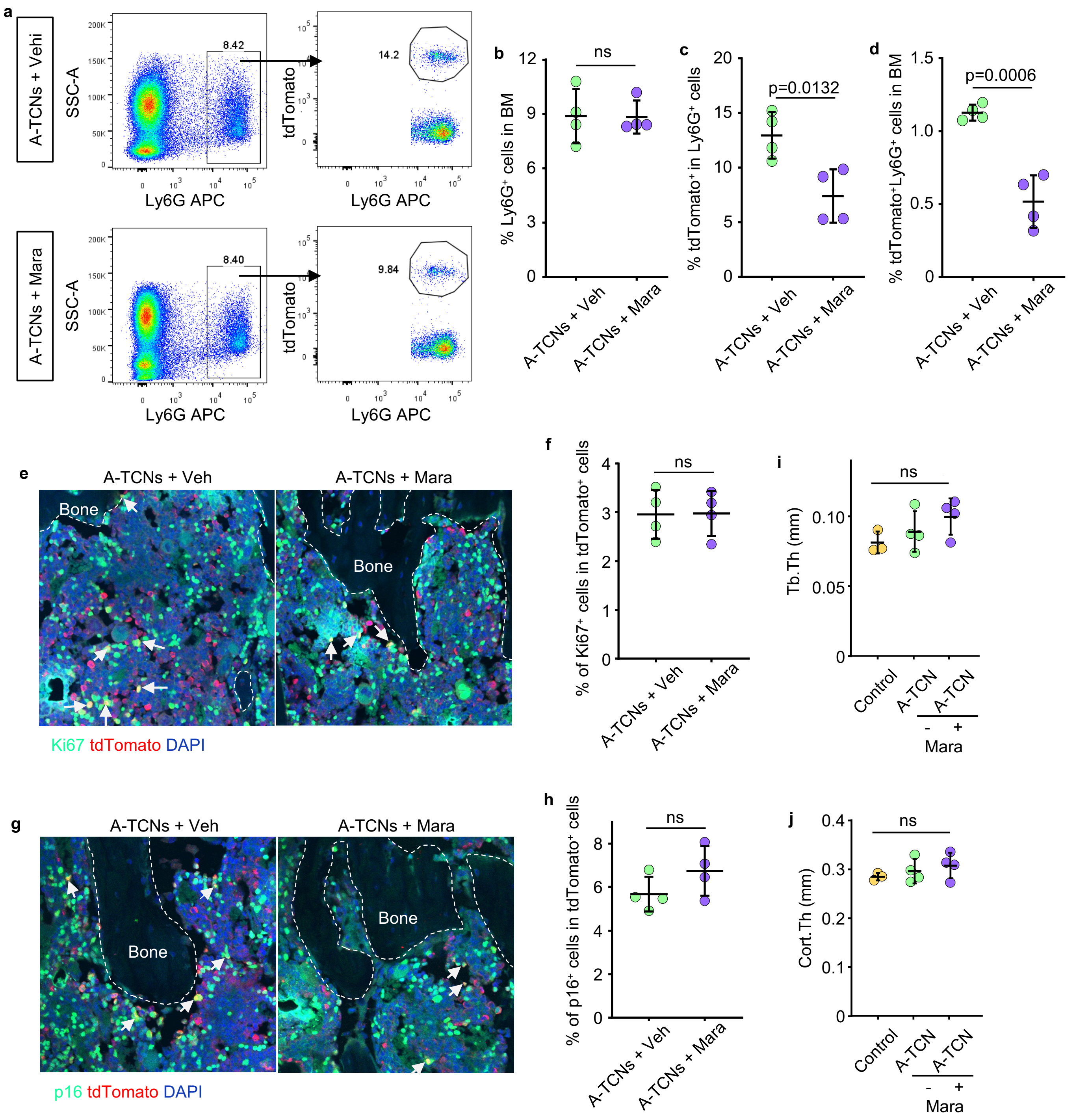

Supplementary Figure 11. Effects of maraviroc on TCN viability and accumulation in bone marrow. (**a**) FACS gates of donor-derived tdTomato$^+$ cells from 20-22-mon-old ROSA26^{mTmG} male mice in total neutrophils (Ly6G$^+$) in BM cells of NSG recipients. (**b-d**) The percentages of Ly6G$^+$ neutrophils in BM cells of recipients (**b**) and of tdTomato$^+$ cells in Ly6G$^+$ neutrophils (**c**) and of tdTomato$^+$Ly6G$^+$ cells in BM cells (**d**) of NSG recipients injected with TCNs from aged ROSA26^{mTmG} mice and treated with vehicle or maraviroc. Mean±SD (n=4 biologically independent male mice/group). (**e**) Cryosections of femoral metaphyses of NSG recipients as in (**a**) stained for Ki-67 (green). A-TCNs from ROSA26^{mTmG} donor mice identified by the expression of tdTomato (red) and donor-derived proliferating cells by both Ki-67 and tdTomato expression (yellow; white arrow), and (**f**) the percentages of Ki-67$^+$ cells in tdTomato$^+$ cells in these stained cryosections. Mean±SD (n=4 biologically independent male mice/group). (**g**) Cryosections of femoral metaphyses of NSG recipients stained for p16 (green) and donor-derived A-TCNs identified by tdTomato expression (red) and donor-derived senescent cells by both p16 and tdTomato expression (yellow; white arrow), and (**h**) the percentages of p16$^+$ cells in tdTomato$^+$ cells. Mean±SD (n=4 biologically independent male mice/group). (**i-j**) Trabecular (**i**) and cortical bone (**j**) thickness of NSG recipients injected with aged mouse-derived TGFβ1$^+$Ly6G$^+$ neutrophils (A-TN), or TGFβ1$^+$CCR5$^+$Ly6G$^+$ neutrophils (A-TCN) plus vehicle or maraviroc. NSG mice without cell injection as control. Mean±SD (n=3, 4 and 4 biologically independent male mice, respectively). Analyses in (**i** and **j**) done using One-way ANOVA with Tukey's post-hoc test and in all others using Student's two-sided unpaired *t* test.

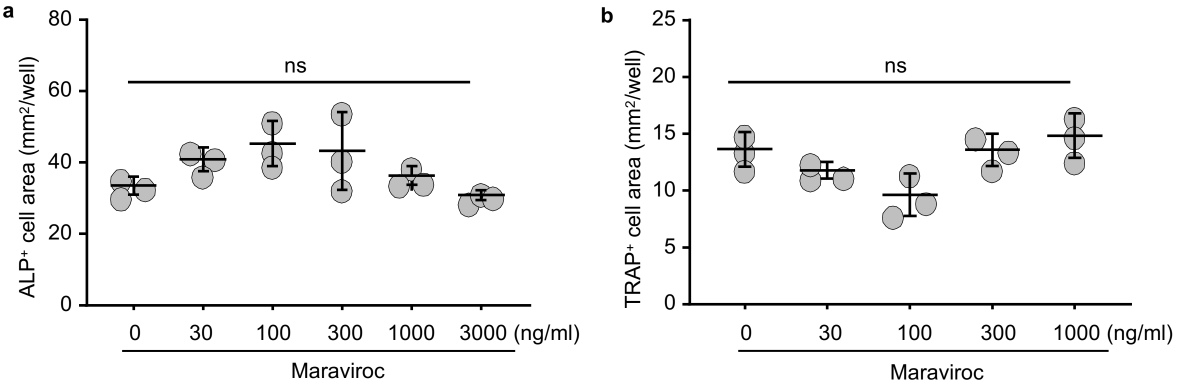

Supplementary Figure 12. Effects of maraviroc on osteoblast and osteoclast differentiation. (a) Bone-derived MPCs seeded in 48-well plates overnight and treated with various concentrations of maraviroc for 4 d. ALP staining for measurement of ALP^+ cell area. (b) BM mononuclear cells seeded in 96-well plates and maintained in medium with addition of M-CSF for 2 d, then treated with 10 ng/ml RANKL plus various concentrations of maraviroc for 2 to 3 d until multinucleated osteoclasts formed. TRAP staining to assess the area of TRAP-positive cells. Mean±SD (n=3 biologically independent samples/group). Analyses: One-way ANOVA with Tukey's post-hoc test.

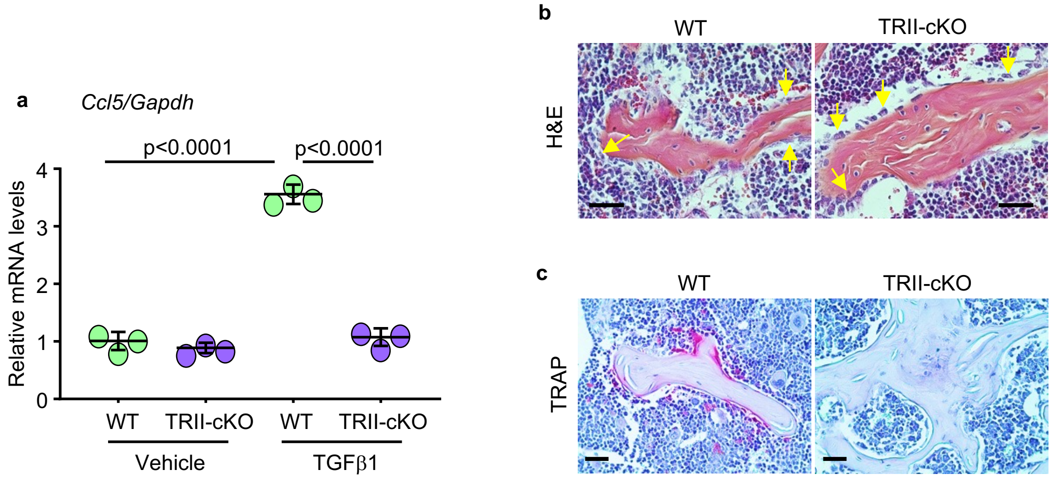

Supplementary Figure 13. TGFβRII deletion in MPCs prevents Ccl5 expression and bone loss. (a) Ccl5 mRNA levels in vehicle- or TGFβ1-treated MPCs from WT and TGFβRII$^{fl/fl}$;Prx1^{Cre} (TRII-cKO) mice. Mean±SD (n=3 biologically independent samples/group). (b) H&E-stained paraffin sections of L3 vertebrae from 15-m-old WT and TRII-cKO mice, and osteoblasts on bone surfaces (indicated by yellow arrows). Bar =20 μm. n=3 female and 5 male biologically independent mice. (c) TRAP-stained paraffin sections of L3 vertebrae from mice in (a). Bar =20 μm; n=3 female and 5 male biologically independent mice. Analyses in (**a**) done using One-way ANOVA with Tukey's post-hoc test.
