# Supplementary material for: TGFβ1+CCR5+ neutrophil subset increases in bone marrow and causes age-related osteoporosis in male mice
Source: Nat Commun. 2023 Jan 11;14:159. doi: 10.1038/s41467-023-35801-z (PMC9834218; doi:10.1038/s41467-023-35801-z)
Supplement: Supplementary file 3 — Reporting Summary [file 41467_2023_35801_MOESM3_ESM.pdf]

nature research

Corresponding author(s): Brendan F. Boyce and Jinbo Li

Last updated by author(s): 2022/12/05

Reporting Summary

Nature Research wishes to improve the reproducibility of the work that we publish. This form provides structure for consistency and transparency in reporting. For further information on Nature Research policies, see Authors & Referees and the Editorial Policy Checklist.

Statistics

For all statistical analyses, confirm that the following items are present in the figure legend, table legend, main text, or Methods section.

n/a	Confirmed	
☐	☒	The exact sample size (*n*) for each experimental group/condition, given as a discrete number and unit of measurement
☐	☒	A statement on whether measurements were taken from distinct samples or whether the same sample was measured repeatedly
☐	☒	The statistical test(s) used AND whether they are one- or two-sided<br>*Only common tests should be described solely by name; describe more complex techniques in the Methods section.*
☒	☐	A description of all covariates tested
☐	☒	A description of any assumptions or corrections, such as tests of normality and adjustment for multiple comparisons
☐	☒	A full description of the statistical parameters including central tendency (e.g. means) or other basic estimates (e.g. regression coefficient) AND variation (e.g. standard deviation) or associated estimates of uncertainty (e.g. confidence intervals)
☐	☒	For null hypothesis testing, the test statistic (e.g. *F*, *t*, *r*) with confidence intervals, effect sizes, degrees of freedom and *P* value noted<br>*Give P values as exact values whenever suitable.*
☐	☒	For Bayesian analysis, information on the choice of priors and Markov chain Monte Carlo settings
☒	☐	For hierarchical and complex designs, identification of the appropriate level for tests and full reporting of outcomes
☒	☐	Estimates of effect sizes (e.g. Cohen's *d*, Pearson's *r*), indicating how they were calculated

Our web collection on statistics for biologists contains articles on many of the points above.

Software and code

Policy information about availability of computer code

Data collection	FACSDiva software was used to collect flow data in FACS LSR II system.
Data analysis	GraphPad Prism 6 was used for data analysis shown in dot graphs. Image Lab 5.1 was used for densitometry analysis of western blot results. FlowJo 7.6.5. was used for flow data analysis. ImageJ 1.8.0 software was used to analyze the fluorescence images. OsteoMeasure was used for bone histomorphometry analysis. Volocity 6.3 Image Analysis Software was used to analyze cell migration velocity.

For manuscripts utilizing custom algorithms or software that are central to the research but not yet described in published literature, software must be made available to editors/reviewers. We strongly encourage code deposition in a community repository (e.g. GitHub). See the Nature Research guidelines for submitting code & software for further information.

Data

Policy information about availability of data

All manuscripts must include a data availability statement. This statement should provide the following information, where applicable:

- Accession codes, unique identifiers, or web links for publicly available datasets
- A list of figures that have associated raw data
- A description of any restrictions on data availability

The raw data for all dot plots are in an Excel file, which is in the Data Source file for this paper along with uncropped blots for all protein bands, and gene expression levels for heatmap generation. Flow cytometry files have benn deposited in FlowRepository with a Repository ID: FR-FCM-Z5VU, which is available following the URL: http://flowrepository.org/experiments/6142.

Field-specific reporting

Please select the one below that is the best fit for your research. If you are not sure, read the appropriate sections before making your selection.

☒ Life sciences ☐ Behavioural & social sciences ☐ Ecological, evolutionary & environmental sciences

For a reference copy of the document with all sections, see nature.com/documents/nr-reporting-summary-flat.pdf

Life sciences study design

All studies must disclose on these points even when the disclosure is negative.

Sample size	The sample size for in vivo experiments is based on an un-paired t-test power analysis carried out by our statistician using SigmaStat Statistical Software: 5–8 mice were needed in each group where bone parameters were being assessed to detect significant differences from controls with an alpha error of 5%. The power is 0.98, i.e., there is 98% chance of detecting a specific effect with 95% confidence when alpha=0.05. Sample sizes for in vitro experiments were based on years of experience using them in what are standard assays.
Data exclusions	No data were excluded from the analyses.
Replication	Each in vitro experiment was repeated at least twice with similar results and all repetitions were successful.
Randomization	Mice were randomly assigned to each treatment group and samples used in in vitro experiments were randomly assigned to each group.
Blinding	Investigators were blinded to group assignment and histomorphometry was done by an investigator who was not involved in the sample collection and group assignment using an OsteoMeasure Image Analysis System (Osteometrics, Decatur, GA) and assessments in immunofluorescence-stained sections presented in Suppl. Fig. 11 done by using ImageJ 1.8.0 software (National Institutes of Health, USA).

Reporting for specific materials, systems and methods

We require information from authors about some types of materials, experimental systems and methods used in many studies. Here, indicate whether each material, system or method listed is relevant to your study. If you are not sure if a list item applies to your research, read the appropriate section before selecting a response.

Materials & experimental systems

n/a	Involved in the study	
☐	☒	Antibodies
☒	☐	Eukaryotic cell lines
☒	☐	Palaeontology
☐	☒	Animals and other organisms
☐	☒	Human research participants
☒	☐	Clinical data

Methods

n/a	Involved in the study	
☒	☐	ChIP-seq
☐	☒	Flow cytometry
☒	☐	MRI-based neuroimaging

Antibodies

Antibodies used

Primary antibodies:
1. Rabbit polyclonal anti-TRAF3, Santa Cruz, sc-947, Clone: M-20, dilution: 1:500;
2. Rabbit polyclonal anti-RelB, Santa Cruz, sc-226, Clone: C-19, dilution: 1:500;
3. Mouse monoclonal anti-GAPDH, Santa Cruz, sc-32233, Clone: 6C5, dilution: 1:500;
4. Rabbit polyclonal anti-p52, CST, 4882s, Clone: N/A, dilution: 1:500;
5. PE/Cy7-conjugated anti-B220, Biolegend, 103222, Clone: RA3-6B2, dilution: 1:100;
6. FITC-conjugated anti-CD45, eBioscience, 11-0451-82, Clone: 30-F11, dilution: 1:100;
7. APC-conjugated anti-CD45, eBioscience, 17-0454-82, Clone: 104, dilution: 1:100;
8. PE/Cy7-conjugated anti-Sca1, eBioscience, 25-5981-81, Clone: D7, dilution: 1:100;
9. PE-conjugated anti-TGFβ1, Biolegend, 141403, Clone: TW7-16B4, dilution: 1:100;
10. APC-conjugated anti-CD11b, eBioscience, 17-0112-83, Clone: M1/70, dilution: 1:100;
11. PE/Cy5.5-conjugated anti-Gr1, eBioscience, 35-5931-80, Clone: RB6-8C5, dilution: 1:100;
12. BV605-conjugated anti-CD115 (CSF1R), Biolegend, 135517, Clone: AFS98, dilution: 1:100;
13. PE/Cy7-conjugated anti-CD11b, eBioscience, 25-0112-82, Clone: M1/70, dilution: 1:100;
14. APC-conjugated anti-Ly6C, eBioscience, 17-5932-82, Clone: HK1.4, dilution: 1:100;
15. APC/Cy7-conjugated anti-Ly6G, Biolegend, 127624, Clone: 1A8, dilution: 1:100;
16. BB515-conjugated anti-CCR5 (CD195), BD, 566208, Clone: C34-3448, dilution: 1:100;
17. PE/Cy7-conjugated anti-CD3e, eBioscience, 25-0031-82, Clone: 145-2C11, dilution: 1:100;
18. Rabbit monoclonal anti-Ki67, Abcam, ab15580, Clone: N/A, dilution: 1:500;
19. Rabbit monoclonal anti-p16INK4a, Abcam, ab241543, Clone: PABLO33B, dilution: 1:400.

Validation

All primary antibodies with satisfactory performance in this study were validated by the supplier in detecting target protein expression in mouse cells and tissue section staining, and additional information including citations can be found on manufacture websites.

1. Primary antibodies provided by Santa Cruz & Cell Signaling Technology:
Abs: 1. Rabbit polyclonal anti-TRAF3, 2. Rabbit polyclonal anti-RelB, 3. Mouse monoclonal anti-GAPDH, 4. Rabbit polyclonal anti-p52. These antibodies were validated by the suppliers for specifically detecting target mouse proteins by Western blot. Citations for these antibodies are readily accessible on the manufacturers' websites by following antibody catalogue information. The performance of these Abs is presented in Fig. 4a and 8a.

2. Primary antibodies provided by eBioscience & Biolegend & BD:
1. PE/Cy7-conjugated anti-B220, 2. FITC-conjugated anti-CD45, 3. APC-conjugated anti-CD45, 4. PE/Cy7-conjugated anti-Sca1, 5. PE-conjugated anti-TGFβ1, 6. APC-conjugated anti-CD11b, 7. PE/Cy5.5-conjugated anti-Gr1, 8. BV605-conjugated anti-CD115 (CSF1R), 9. PE/Cy7-conjugated anti-CD11b, 10. APC-conjugated anti-Ly6C, 11. APC/Cy7-conjugated anti-Ly6G, 12. BB515-conjugated anti-CCR5 (CD195), 13. PE/Cy7-conjugated anti-CD3e. These antibodies were validated by the suppliers by specifically detecting target mouse proteins via flow cytometry. Citations for these antibodies are readily accessible on the manufacturers' websites by following antibody catalogue information. The performance of these Abs is presented in Fig. 1c/d/e, Suppl. Fig. 1a/2a/3a&b/8a&b/9a/10a&b/11a.

3. Primary antibodies provided by Abcam:
1. Rabbit monoclonal anti-Ki67, Abcam, 2. Rabbit monoclonal anti-p16INK4a. These antibodies are validated by the suppliers in detecting Ki67 and p16 by IF. Citations for both antibodies can be found on the manufacturer's website by following antibody catalogue information. The data on these Abs are presented in Suppl. Fig. 11e&g.

Animals and other organisms

Policy information about studies involving animals; ARRIVE guidelines recommended for reporting animal research

Laboratory animals

All animal procedures were conducted in compliance with all applicable ethical regulations using procedures approved by the University of Rochester Committee for Animal Resources. All mice used in this study were appropriately monitored under the University Committee on Animal Resources of the University of Rochester guidelines with the Animal Welfare Assurance number D16-00188 (A3292-01). CO2 inhalation was used for mouse euthanasia. Briefly, no more than 5 mice were placed in a transparent euthanasia chamber (7½" wide x 11½" deep x 5" high) and CO2 was introduced at 3.0 l/min. Once the mice appeared unconscious (i.e., recumbent, without purposeful movement), the CO2 influx speed was increased to 5.0 l/min. All mice were left in the CO2 environment for at least 5 min until breathing and heartbeat stopped, and they had faded eye color, fully dilated pupils, and relaxation of anal/urinary sphincters.

1. Mice with TRAF3 conditionally knocked out in osteoblast (OB) lineage cells were generated by crossing TRAF3flox/flox mice (C57BL/6 background) with Prx1Cre mice (Jackson Lab; Stock #: 005584; which we call P-cKO mice). P-cKO mice and their WT (TRAF3flox/flox) littermates were sacrificed at 12 mon of age.

2. Mice with TGFβRII conditionally knocked out in osteoblast lineage cells were generated by crossing TGFβRIIflox/flox mice (Jackson Lab #012603) with Prx1Cre mice (Jackson Lab #005584). TGFβRIIflox/floxPrx1Cre mice (which we call T-cKO mice) and their WT (Traf3flox/flox) littermates were sacrificed at 15 months of age.

3. ROSAmT/mG mice (Jackson Lab #007676) have cell membrane-localized tdTomato (mT) fluorescence expressed widely in cells/tissues prior to Cre recombination, while, in Cre recombinase-expressing cells (and future cell lineages derived from these cells), cell membrane-localized EGFP (mG) fluorescence expression will be triggered and replace the red fluorescence. To examine the specificity of targeted gene deletion driven by Prx1Cre, we crossed Prx1Cre with Rosa26mTmG reporter mice, in which Prx1Cre+ cells switch tdTomato to GFP protein expression. On 3-mon-old Prx1Cre;Rosa26mTmG mice, a non-stabilized tibia fracture model was performed after mice being anesthetized by isoflurane. Briefly, an incision was made along the anterior side of the mouse tibia, and a transverse osteotomy at the mid-shaft of the mouse tibia was unilaterally performed using a rotary bone saw, and the incision was closed without fixation of fractured bones. Fractured bones were harvested on day14 post-fracture for further analysis.

4. WT C57 mice were purchased at breeding age from the National Cancer Institute (Frederick, MD, USA) and bred to generate young WT mice that were sacrificed at 2-3 months of age. Aged C57 mice (22-mon-old) were provided by the National Institute on Aging (Bethesda, MD, USA).

All these mouse lines are in a C57BL/6 (C57) background. We used male mice in all experiments except those used in Figure 8 (in which 3 pairs of female TGFβRII-cKO and WT mice were used) to avoid the effects of changing levels of female sex hormones on bone mass and metabolism during aging.

Mice were housed with a 12/12 light cycle (typically 6 am ON and 6 pm OFF) at 720 F (+/- 2 degrees) and 30% to 70% humidity.

Wild animals

No wild animals were used in the study.

Field-collected samples

No field collected samples were used in the study.

Ethics oversight

All animal procedures were conducted in compliance with all applicable ethical regulations using procedures approved by the

Ethics oversight | University of Rochester Committee for Animal Resources.

Note that full information on the approval of the study protocol must also be provided in the manuscript.

Human research participants

Policy information about studies involving human research participants

Population characteristics | The study enrolled 55 subjects, including 28 females and 27 males, ranging from 8- to 87-years-old, of which 26 subjects were 8–18-year-old children (10 males, 16 females) and the remaining 29 were middle-aged to elderly from 53 to 87 years (18 males, 11 females). Subjects with tumors, active systemic, immunologic, inflammatory, or metabolic disorders that might affect bone remodeling were excluded.

Recruitment | We collected samples of vertebral bone that were removed from pediatric and adult patients undergoing elective surgery to correct spinal scoliosis and degenerative conditions, including cervical spondylosis, lumbar spinal stenosis, and disc herniation.

Ethics oversight | We followed a protocol with informed consent from all patients or their guardians in the case of pediatric patients that was approved by the Research Subjects Review Board of the University of Rochester Medical Center. These human studies were performed with adherence to the relevant ethical regulations (Declaration of Helsinki).

Note that full information on the approval of the study protocol must also be provided in the manuscript.

Flow Cytometry

Plots

Confirm that:

☒ The axis labels state the marker and fluorochrome used (e.g. CD4-FITC).

☒ The axis scales are clearly visible. Include numbers along axes only for bottom left plot of group (a 'group' is an analysis of identical markers).

☒ All plots are contour plots with outliers or pseudocolor plots.

☒ A numerical value for number of cells or percentage (with statistics) is provided.

Methodology

Sample preparation | BM was flushed out with FACS buffer (2% FBS/PBS) from leg bones dissected from young and old C57 mice that had been treated with vehicle or maraviroc, as well as P-cKO and TRII-cKO mice and their respective WT littermates.

Instrument | BD FACS LSRII analyzing cytometer and BD FACSAria II sorting cytometer

Software | FlowJo software was used for data analysis.

Cell population abundance | The purity of TCNs in post-sort fractions was over 90%, which was confirmed using BD FACS LSRII cytometer. Positively-selected cells were confirmed to be Ly6G+ cells (>96%) using FACS LSRII.

Gating strategy | We first gated BM cells using a “J”-shaped gate and to exclude DAPI-positive dead cells and then used forward scatter height (FSC-H) versus forward scatter area (FSC-A) plot, and side scatter height (SSC-H) vs side scatter area (SSC-A) plot to exclude doublets. To further analyze TCNs, TGFbeta1-positive BM cells were gated out basing on full-minus-one (FMO) control, and CD11b-positive fraction (>90%) were next gated out from TGFbeta1+ BM cells, and then Ly6C and Ly6G expression on TGFbeta1+CD11b+ BM cells were analyzed before measurement of CCR5 expression (Suppl. Fig. 8).

☒ Tick this box to confirm that a figure exemplifying the gating strategy is provided in the Supplementary Information.
