# Supplementary material for: TGFβ1+CCR5+ neutrophil subset increases in bone marrow and causes age-related osteoporosis in male mice
Source: Nat Commun. 2023 Jan 11;14:159. doi: 10.1038/s41467-023-35801-z (PMC9834218; doi:10.1038/s41467-023-35801-z)
Supplement: Supplementary file 4 — Source Data [file 41467_2023_35801_MOESM4_ESM.zip › Source data/Inventory of Supporting Information.docx]

Inventory of Supporting Information

1. Suppl.Figs.
2. Primers for chemokine genes-Fig. 5a
3. Original data for all dot plots
4. Original blots
5. Information about Abs and reagents
6. Heatmap-Log2-Fig. 5a
