# Supplementary figures and images for: TGFβ1+CCR5+ neutrophil subset increases in bone marrow and causes age-related osteoporosis in male mice
Source: Nat Commun. 2023 Jan 11;14:159. doi: 10.1038/s41467-023-35801-z (PMC9834218; doi:10.1038/s41467-023-35801-z)

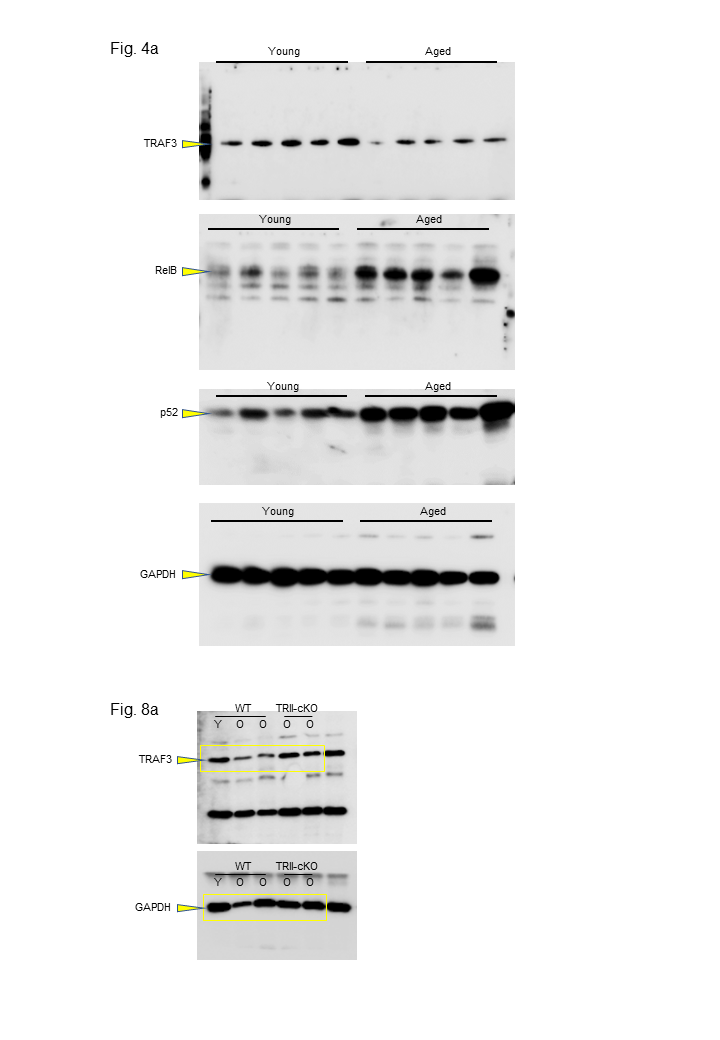

Supplement: Supplementary file 4 — Source Data [file 41467_2023_35801_MOESM4_ESM.zip › Source data/Original blots-2022 NC resubmission.TIF]
